# Composting of common organic wastes using microbial inoculants

**DOI:** 10.1007/s13205-011-0033-5

**Published:** 2011-11-17

**Authors:** Ieshita Pan, Bomba Dam, S. K. Sen

**Affiliations:** Microbiology Division, Department of Botany, Visva-Bharati, Santiniketan, 731235 India

**Keywords:** Compost, C:N ratio, NH_4_-N, NO_3_-N

## Abstract

It is important to use renewable resources to maximize crop yields and minimize the environmental hazards associated with chemical residues. Composting is an age old practice for the biological conversion of organic waste to a humus-like substance which can enhance physical, chemical and biological soil properties. To explore the effect of microorganisms in the composting process, three potent bacterial isolates were selected. Their morphological, cultural and biochemical characteristics were identified, and 16S rDNA studies identified isolates B1U/1 and D3L/1 as *Bacillus subtilis* and isolate RAT/5 as *Pseudomonas* sp. Common organic wastes were composted using the selected isolates individually and as a consortium. The C/N ratio of each substrate reduced gradually to 25–30:1 within 120 days and remained constant thereafter. The reduction in NH_4_^+^ and NO_3_^−^ ion concentrations also indicated compost maturity after 120 days. The pH of the mature compost was typically 7.0 ± 0.2, and the PO_4_^−3^ ion concentration was high throughout the decomposition process. This study describes the optimization of the composting process using a consortium of isolates from composted soil.

## Introduction

Environmental contamination has the potential to be a major threat to the survival of living organisms. The misuse of chemical fertilizers and pesticides can contribute to the deterioration of the environment (Kaosol 2009). Population migration to urban areas and urban development can also lead to the depletion of fossil fuels, generation of carbon dioxide and contamination of water resources, all of which can lead to environmental contamination. Contamination can affect soil fertility resulting in a loss of productivity, and this realization has led to the adoption of sustainable farming practices with the aim of reversing the declines in productivity and environmental protection (Wani et al. 1995; Gautam et al. 2010).

In India, nearly 700 million tons of organic waste is generated annually, leading to challenges for its safe disposal, with the waste being usually either burned or land filled (Bhiday 1994; Nagavallemma et al. 2006; Zeinhom et al. 2010). However, there are several naturally occurring microorganisms that are able to convert organic waste into valuable resources such as plant nutrients, and reduce the C:N ratio to support soil productivity. These microorganisms are also important to maintain nutrient flows from one system to another and to minimize ecological imbalance (Novinsak et al. 2008; Umsakul et al. 2010).

Composting is a preferred and environmentally sound method whereby organic waste is reduced to organic fertilizer and soil conditioners through biological processes (Gautam et al. 2010; Alexander 1999). The high organic carbon content and biological activity of compost make it effective for applications such as erosion control and revegetation (Anastasi et al. 2005). The composting process involves three phases, and uses diverse microflora such as bacteria, fungi and mesophilic (*Streptomyces rectus*) and thermophilic *Actinomycetes* (*Actinobifida chromogena* (*Thermomonospora fusca*) *Microbispora* (*Thermopolyspora*) *bispora, Therinomnonospora curvata, Thermoactinomyces* sp.) eventually converting organic waste to humus (Buyuksonmez et al. 2000; Pedro et al. 2003; Schloss et al. 2003; Zeng et al. 2011). During the first phase there is an increase in carbon dioxide along with the temperature. The substrate is reduced due to the degradation of sugar and proteins by the action of mesophilic organisms (Hellmann et al. 1997; Schloss et al. 2003; Novinsak et al. 2008; Zeng et al. 2011). The second phase leads to an increase of the temperature in the compost piles from 45 °C to approximately 70 °C and the mesophiles are replaced by thermophiles (Pedro et al. 2003; Schloss et al. 2003). Large numbers of pathogenic individuals are degraded during this time (Novinsak et al. 2008). The third phase begins with the decrease of temperature of the compost pile.

The quality and stability of compost is entirely dependent on its raw materials (Ranalli et al. 2001; Benito et al. 2003; Wang et al. 2004). During the composting process, various parameters including the C:N ratio, composting temperature, pH of the finished product, moisture content, and the presence of potential pathogens such as coliform bacteria are used to assess the quality and stability of the compost (Wu and Ma 2002; Steger et al. 2007; Erickson et al. 2009; Al-Turki 2010; Fourti et al. 2011; Sanmanee et al. 2011).

This paper monitors the decomposition of common household organic waste with a microbial consortium to identify a suitable composting method. In particular, this paper focuses on the identification of suitable but cheap raw materials, processes that use minimal energy, and the selection of proper microorganisms to produce quality compost.

## Materials and methods

### Organism maintenance and preparation

Three bacterial isolates, *Bacillus subtilis* B1U/1, *B. subtilis* D3L/1 and *Pseudomonas* sp. RAT/5 were selected from more than two hundred isolates from diverse compost samples. The hydrolysers, *Bacilli* isolates B1U/1 and D3L/1, were maintained on slants of medium containing 5.0 gL^−1^ carboxy methyl cellulose, 2.0 gL^−1^ NaNO_3_, 1.0 gL^−1^ K_2_HPO_4_, 0.5 gL^−1^ MgSO_4_·7H_2_O, 0.5 gL^−1^ KCl, 2.0 gL^−1^ peptone, and 15.0 gL^−1^ agar, held at pH 7.0 and 4 °C. The nitrogen fixer, *Pseudomonas* isolate RAT/5 was maintained in a mannitol nitrogen free agar medium containing 15.0 gL^−1^ mannitol, 0.5 gL^−1^ K_2_HPO_4_, 0.2 gL^−1^ MgSO_4_·7H_2_, 0.1 gL^−1^ CaSO_4_, 0.2 gL^−1^ NaCl, 5.0 gL^−1^ CaCO_3_, and 15.0 gL^−1^ agar held at pH 7.3.

To achieve successful and rapid composting, inoculation by bacteria was needed. The sterilized raw materials were inoculated with 2% broth inoculum (10^9^ CFU/mL) of each of the isolates, individually. To check the combined activity of the isolates, a 1% broth inoculum containing 10^7^ CFU/mL of each isolate was used.

The hydrolytic potentials of the isolates B1U/1, D3L/1 and RAT/5, particularly the cellulose, amylase (Miller 1959) and protease (Anson 1938) activities were measured, and protein estimations were performed (Lowry et al. 1951). All organisms were checked for their nitrogen fixation ability by growing them in nitrogen free medium.

### Composting process

Seven raw materials (common organic wastes), fruit wastes, vegetable wastes, leaves, hay, newspaper, wheat straw and rice husks, identified as substrates C1–C7, respectively, were used for the composting experiment, as the nature of the raw material directly affects the quality of final product (Lasaridi and Stentiford 1996). Initially 20 g of each waste substrate was used in the experiments. All substrate samples were sterilized and inoculated with *B. subtilis* B1U/1, *B. subtilis* D3L/1 or *Pseudomonas* sp. RAT/5, both separately and in a consortium. The inoculated flasks were maintained at room temperature (30 ± 2 °C) and 45–50% moisture during composting. The composting process was conducted in triplicate and monitored for up to 6 months.

### Sampling and analysis

Three sub-samples (1 g each) were taken at day 1 of the composting process and every 15 days thereafter (Gillet 1986). The first sub-sample was stored at 4 °C to provide a sample library; the second sub-sample was used for the physicochemical analyses; and the third sub-sample was used for the microbiological analyses.

At each sampling period, physical characteristics including color by visual observation, moisture content by gravimetric analysis, odor by olfactory analysis and texture by coarseness evaluation, were determined.

At each sampling period, the pH was determined using pH meter (1:10, water:extract) and moisture content was calculated by a simple deduction of water loss. The chemical composition of the sub-samples was determined, including the organic carbon (Black 1965), nitrogen (Vogel 1961), phosphate (Jackson 1968) and potassium (Wen et al. 1997) concentrations.

### Statistical analysis

All experiments were conducted in triplicate. The values reported in this paper are mean ± SD (Snedecor and Cochran 1980).

## Results and discussion

### Organism selection and characterization

Three isolates were selected based on their hydrolytic potentials from 200 isolates in a diverse soil habitat, for this decomposition study. The selected organisms were repeatedly tested for their ability to produce extracellular cellulase, amylase and protease, as Umsakul et al. (2010) reported that organisms involved in organic decomposition processes were able to produce high levels of hydrolytic enzymes.

Isolates B1U/1 and D3L/1 were identified as members of the *Bacillus* genus while isolate RAT/5 was identified as a *Pseudomonas* sp. based on their morphological and cultural characteristics and biochemical properties (data not shown). For the molecular characterization, nearly complete 16S rRNA gene sequences were determined for isolates B1U/1 (1,411 bp) and RAT/5 (1,370 bp) and these were compared with the non-redundant nucleotide database at the National Center for Biotechnology Information (NCBI) (Pearson and Lipman 1988). In a sequence analysis, isolates B1U/1 (Accession No. GU723510) and D3L/1 (Accession No. GU723508) showed a 100% similarity with *B*. *subtilis* strains while isolate RAT/5 (Accession No. GU723511) showed a 99% similarity with *Pseudomonas* sp. bacteria, with the *Escherichia, Klebsiella, Aeromonas, Pseudomonas, Alcaligenes, Bacillus* and *Enterococcus* genera most likely (Ishii et al. 2000). *Pseudomona*s sp. RAT/5 was able to grow in the complete absence of nitrogen, and was, therefore, classified as an atmospheric nitrogen fixer.

### Physical characteristics

During the composting process, gradual changes of the textures of the raw materials were observed after 30 days, followed by the appearance of a black colored humus-like substance which developed after 120 days of decomposition. In this study, only wheat straw (substrate C6) was converted into compost within 75 days. No textural change was observed for the newspaper waste material.

The moisture content was allowed to reduce after 90 days, while the optimum amount of decomposition occurred after 120 days, and the process was monitored for up to 6 months. The moisture content was maintained at 40–60% by sequential watering to replace any water loss. Moisture appeared to be a key influencing factor for microbial activity (Anastasi et al. 2005), as low moisture contents inhibit the growth of beneficial microorganisms (Umsakul et al. 2010), while excess moisture can create anaerobic conditions, leading to the production of unpleasant odors and toxic volatile substances (Saidi et al. 2008).

Weight loss was observed during the compost formation process. The weight losses were calculated using a simple deduction method. The reduction in weight was more significant during the first 45 days, which is a similar result to those observed by Andrea et al. (1998), who measured a weight loss of 29%, and Gautam et al. (2010) who observed weight loss over a 45-day period.

### Chemical characteristics

#### Nitrogen transformation during composting

**Fig. 1:**
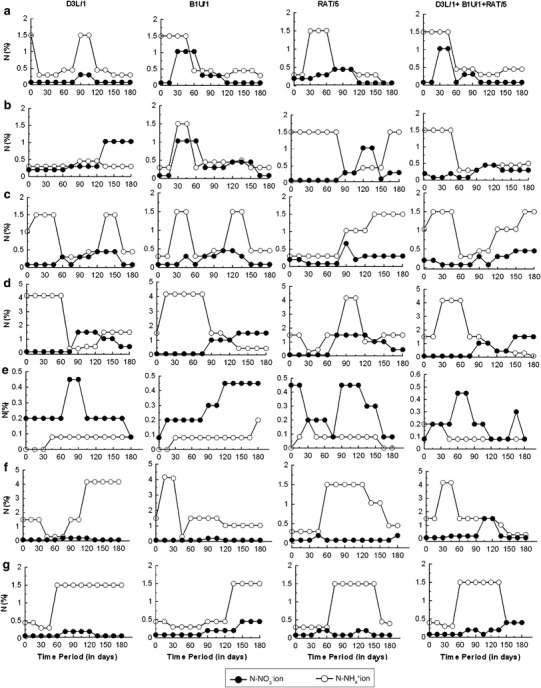
Changes in nitrogen composition during the decomposition of different waste substrates: **a** fruit waste; **b** vegetable waste; **c** leaves; **d** hay; **e** newspaper waste; **f** wheat straw; **g** rice husks. Results are the mean value from three independent experiments

The NH_4_-N concentration decreased for all substrates at different rates. The NH_4_-N content in the different substrates ranges from 0.2 to 4.17%, while the NO_3_-N ranges from 0.1 to 0.5%. After 60 days of decomposition, variations were observed in the NH_4_-N and NO_3_-N concentrations (Fig. 1). Figure 1 also shows that for the substrate C2 (vegetable waste) with isolate B1U/1 and C5 (news paper waste) with isolate RAT/5, NH_4_-N concentration increased during the first 55 days followed by a sharp decrease, with a steady NH_4_-N concentration after 90 days. However, for substrate C3 (leaves) inoculated with isolate RAT/5, C5 (newspaper waste) with isolate B1U/1, and C7 (rice husks) with isolates B1U/1 and D3L/1, a continuous increase in the NH_4_-N concentration was observed throughout the composting process. At the completion of the composting process, for all seven substrates, the relative proportions of the nitrogenous species (NH_4_^+^ N and NO_3_^−^ N) indicate that ammonia was converted to nitric acid by the following process, as also observed by Saidi et al. (2008).

$$ {\text{NH}}_{ 4}^{ + } + 2 {\text{O}}_{ 2} \to {\text{ NO}}_{ 3} + {\text{ 2H}}^{ + } + {\text{ H}}_{ 2} {\text{O}} $$

$$ 2 {\text{H}}^{ + } + {\text{ 2NO}_{ 3}^{ - }} \to {\text{ 2HNO}}_{ 3} $$

#### C/N ratio

**Fig. 2:**
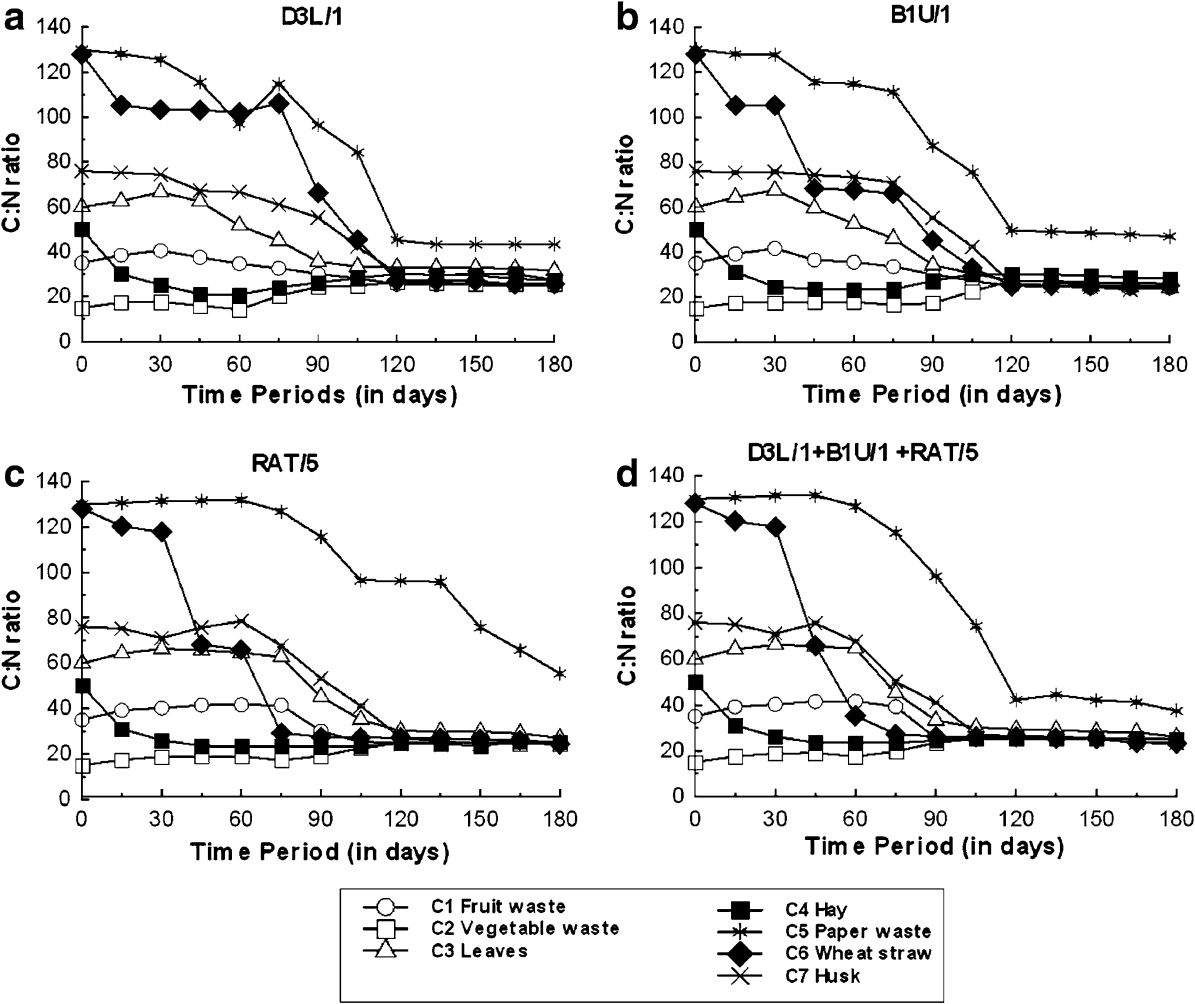
Change in C:N ratio for different waste substrates during composting. Results are the mean value from three independent experiments

The initial organic carbon content was relatively high for each substrate, at between 20 and 80%. The initial C:N ratios were 35:1 (C1; fruit waste), 15:1 (C2; vegetable waste), 60:1 (C3; leaves), 50:1 (C4; hay), 125:1 (C5; newspaper waste), 128:1 (C6; wheat straw), and 76:1 (C7; rice husks), which is consistent with the observations of Hadas and Portnoy (1994). The C:N ratio gradually decreased for all substrates except C2, for which the C:N ratio increased for the first 60 days and then remained steady (Fig. 2). The overall nitrogen loss during later stages of the composting process caused the increase in this C:N ratio. Atkinson et al. (1996) reported that a reduction of 29% of the organic carbon content occurs during composting of organic waste; while a reduction of only 10% in the carbon content was estimated by Erickson et al. (2009) and Umsakul et al. (2010). In this study, the C:N ratio increased in some cases during the first 30 days of decomposition followed by a sharp decrease and was stable after 120 days, although this pattern varied depending on the test organism. When the consortium of three isolates (1:1:1) was used, the rate of decomposition was faster and the C:N ratio reduced to around 25–30:1 at 75–90 days, depending on the substrate. The only exception was for C5 (newspaper waste) which required more than 180 days for the decomposition (Fig. 2d). Of the seven substrates tested, C6 (wheat straw) had a greatest increase in the rate of decomposition while using the bacterial consortium (Fig. 2d).

The decrease in the C:N ratio can be explained by the transformation of organic carbon into carbon dioxide, followed by a reduction in the organic acid content (Chefetz et al. 1998; Sanmanee et al. 2011). Saidi et al. (2008) reported that a stable C:N ratio could be achieved after 95 days of decomposition. High C:N ratios can indicate the presence of unutilized complex nitrogen substrates (Saidi et al. 2008; Fourti et al. 2011), while low C:N ratios (less than 20:1) indicate the instability of the compost (Haug 1993). The completion of the composting process (compost maturity) is indicated when the C:N ratio reduces to between 25 and 30:1 (Fig. 2d) (Hardy et al. 1993).

#### pH changes during composting

**Fig. 3:**
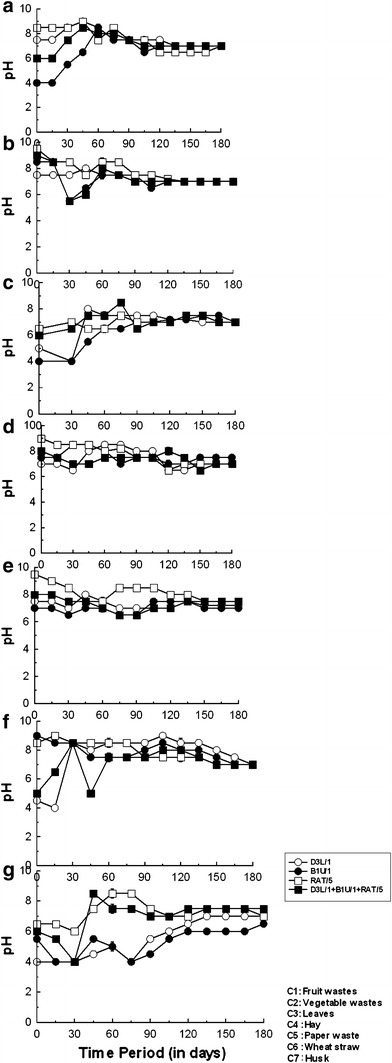
pH change during composting of different waste substrates: **a** fruit waste; **b** vegetable waste; **c** leaves; **d** hay; **e** newspaper waste; **f** wheat straw; **g** rice husks. Data are the mean of three independent experiments

The changes in pH change during the composting process are shown in Fig. 3. The initial pH values were between 4.0 and 9.0, depending on the substrate. In the first 60 days of composting, a pH increase was observed for substrates C3 (leaves; inoculated with B1U/1) and C1 (fruit waste; inoculated with the consortium). The pH increase is the result of volatilization and microbial decomposition of organic acids, and the release of ammonia by microbial mineralization of organic nitrogen sources (Mckinley and Vestal 1985). For substrates C2 (vegetable waste) and C4 (hay) inoculated with the consortium, and C6 (wheat straw) with RAT/5, an initial drop in the pH was recorded in the first 30 days. A similar pH drop was observed by Poincelot (1974), and White et al. (1995) suggested that an alkaline pH could enhance the composting process, controlling pathogenic fungi that prefer acidic growth conditions (Saidi et al. 2008). The decomposition of organic wastes at pH values of 6.0 or below can slow down the decomposition process, while pH values above 8.0 can cause the release of unpleasant smells associated with ammonia. Earlier studies have identified that microbial activity enhanced the likelihood of achieving a suitable pH range of 5.5–9.0; while the composting process is most effective at pH values between 6.5 and 8.0 (Christian et al. 1997). The pH value stabilized at close to 7.0 after 120 days of composting for all substrates, except the wheat straw (Fig. 3d), which achieved a neutral pH after at 90 days.

#### Phosphate ion concentration

**Fig. 4:**
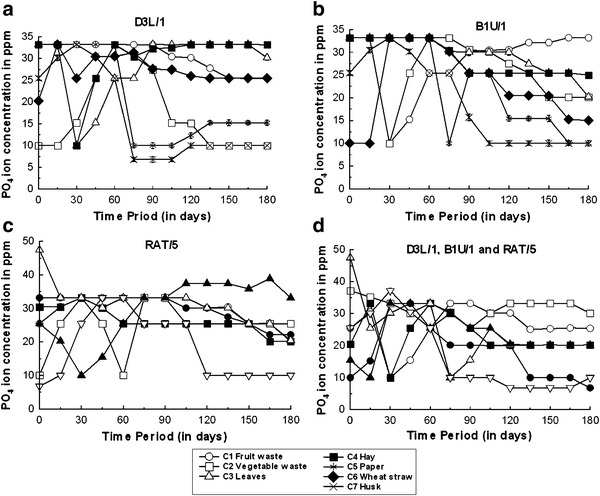
Changes in the phosphate ion concentration during composting. Data are the mean value of three independent experiments

Throughout the composting process, the phosphate ion concentration varied between 6.8 and 47.46 mg/kg, depending on the substrate and inoculum (Fig. 4). The lowest phosphate ion concentrations were measured for substrate C7 (rice husks; inoculated with isolate D3L/1, Fig. 4a), while the maximum concentration was measured for substrate C5 (newspaper waste; inoculated with *Psudomonas* sp RAT/5, Fig. 4c). The phosphate ion concentration remained constant for substrates C1 (fruit waste; with *B. subtilis* D3L/1, Fig. 4a) and C4 (hay; with *B. subtilis* B1U/1, Fig. 4b). Dinel et al. (2004) reported that nitrogen deficiency of the substrate could be improved by the addition of phosphoric acid, which could also prevent the excessive volatilization of ammonia. Phosphorus is sometimes applied during composting to maintain a C:P ratio between 75:1 and 150:1 (Taiwo and Oso 2004; Gautam et al. 2010). A high percentage of the phosphate present in compost is available during the plant growing season, but nutrient availability depends on the quality of soil, particularly its moisture content and temperature (Hue et al. 1994; Wen et al. 1997), and the ready availability of phosphate enhances soil carbon and nutrient uptakes (Yadav et al. 1999).

#### Statistical analysis

Statistical analysis of all the biological parameters revealed that the consortia decomposed the raw material within a very short time period. However, among the different raw materials, wheat straw decomposed rapidly to correlate between microbial colonization and rate of decomposition.

## Conclusion

There are many efficient hydrolytic bacteria and other physiologically important microorganisms present in compost. Several isolates from the *Bacilli* and *Pseudomonas* genera were selected based on their hydrolytic potentials for this study. The isolates were applied as a starter culture in the composting of various organic waste substrates, and a consortium of the inoculants (in a 1:1:1 ratio) was able to efficiently decompose all the substrates tested. The chemical composition of end products, even from the initial experimental stage, was consistent with national and international standards for composting (Hogg et al. 2000).

For successful composting, the selection of the most appropriate raw material is an important component (Fourti et al. 2011). Of the substrates tested, wheat straw was the most suitable material for large scale composting using the bacterial consortium, and this substrate is readily available and very cheap. With the consortium including both cellulolytic and nitrogen fixing bacteria, the rate of decomposition with wheat straw was maximized the compost was stable after 75 days, with a pH value of 7.0 ± 0.2 and a C:N ratio close to 25:1. The experimental results indicate that the consortium is more effective than any individual isolate. The data show enough promise in the sustainable production of organic fertilizer using the consortium to instigate a pilot plant experiment.
